# RES6/466: Toward a Discovery Support System Based on Medical and Health Unifying Principles to Formulate Recombinant Hypotheses through Internet Online Databases

**DOI:** 10.2196/jmir.1.suppl1.e81

**Published:** 1999-09-19

**Authors:** R.J Stusser

**Affiliations:** ^1^Clinical Research CentreHavanaCuba

**Keywords:** Unifying Principles, Inductive Method, Hypothesis Formulation, Internet, Discover Support System

## Abstract

**Introduction:**

Since the 17-century, scientists have been enquiring for the logical scientific principles of medicine and informatics, among other disciplines, encouraged by the instance of Newtonian physics. In the 20-century, the main principles of informatics were found making possible the development of present computers & Internet. However, very little research has been done seeking medical & health scientific principles, allowing among other functions, assistance in scientific hypotheses formation beside empirical data. One important effort on hypothesis formulation, has been the running of the Arrowsmith system of software and database search strategies at http://kiwi.uchicago.edu (Swanson & Smalheiser, 1997), which evokes hypothesis using the relational structure of the NCBI PubMed Internet on-line database (1966-). Nevertheless, although it uses a powerful logical mathematical method, it does not include any logical scientific principle from experimental or clinical medicine, & public health sciences. The aim of this paper is to give an outline of the design & rationale of an international collaborative research, complementary to Arrowsmith system, whose outcomes would be the logical basis of content seeking a more rational discovery support system.

**Methods:**

Crucial fragmented information of multiple specialities and cognitive levels, synthesised by an international cross-disciplinary team or teams of experts, through a complex inductive method using Internet research facilities.

Expected Results:

Medical & health unifying principles that would perfect Arrowsmith target search strategies or other formal discovery computer-assisted systems to formulate recombinant hypotheses, using PubMed on-line database, and even in the future, the NCBI E-Biomed Internet on-line database proposed at http://www.nih.gov/welcome/director/ebiomed/ebiomed.htm (Varmus, Lipman & Brown, 1999). The perfected system will complete then, the premises to receive the benefits of Artificial Intelligence concepts & tools, to continue its improving.

